# Induction of Pyroptosis and Its Implications in Cancer Management

**DOI:** 10.3389/fonc.2019.00971

**Published:** 2019-09-26

**Authors:** Yan-Yang Wang, Xin-Lan Liu, Ren Zhao

**Affiliations:** ^1^Department of Radiation Oncology, General Hospital of Ningxia Medical University, Yinchuan, China; ^2^Cancer Institute, Ningxia Medical University, Yinchuan, China; ^3^Department of Medical Oncology, General Hospital of Ningxia Medical University, Yinchuan, China

**Keywords:** pyroptosis, gasdermin D (GSDMD), gasdermin E (GSDME), cancer progression, therapeutic sensitivity

## Abstract

Pyroptosis is a gasdermins mediated programmed cell death, which has been widely studied in inflammatory disease models. Recently, there are growing evidences that pyroptosis can be chemically induced in cancer cells without any bacterial or viral infection. Pyroptosis may affect all stages of carcinogenesis and has become a new topic in cancer research. In this review, we first briefly introduced pyroptosis. In the subsequent section, we discussed the induction of pyroptosis in cancer and its potential role as a promising target for cancer therapy. In addition, the biological characteristics of gasdermin D (GSDMD) and gasdermin E (GSDME), two important pyroptosis substrates, and their prognostic role in cancer management were reviewed. These results help us to understand the pathogenesis of cancer and develop new drugs, which based on pyroptosis modulation, for cancer patients.

## Introduction

Inflammation is one of the hallmarks of cancer (1). Inflammasomes are the most critical components of the response to cancer promoting inflammation (2–4). Once activated by diverse danger signals of pathogenic or non-pathogenic origin, inflammasomes can trigger the maturation and secretion of pro-inflammatory cytokines, such as interleukin-1β (IL-1β) and IL-18, to influence the pathogenesis of cancer by modulating innate and adaptive immune responses (5–7). Strong associations between dysregulation of inflammasomes and malignant diseases highlight the importance of this pathway in cancer management (8).

Pyroptosis, the inflammasomes-induced programmed cell death mediated by gasdermins, is first described in myeloid cells infected by pathogens or bacteria in 1992 (9). Pyroptosis is thought to play a key role in the clearance of various bacterial and viral infections by removing intracellular replication niches and enhancing the host's defensive responses (10). Dysregulation of pyroptosis may cause lower efficiency of pathogens clearance and dysfunction in the stimulation of adaptive immune defenses, resulting in tissues damage (11). More recently, growing evidences demonstrated that pyroptosis could be chemically induced in cancer cells without any bacterial or viral infection (12). Pyroptosis has become a new topic in cancer research because it may affect all stages of carcinogenesis. Advances on the morphological characteristics and mechanisms of pyroptosis will broaden our understanding of cancer and provide new perspectives in cancer management (13, 14).

In this mini-review, we firstly give a brief introduction of pyroptosis. The activation of pyroptosis in cancer and its prognostic role in cancer management will be discussed subsequently.

## Overview of Pyroptosis

Pyroptosis is a form of programmed cell death, which is featured by cell membrane pore formation, cytoplasmic swelling, membrane rupture and the release of cytosolic contents such as IL-1β into the extracellular environment, amplifying the local or systemic inflammatory effects (15, 16).

**Figure 1:**
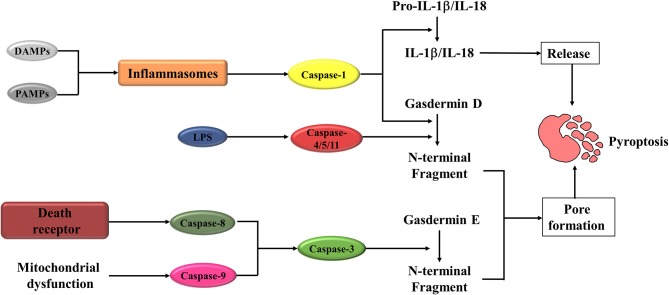
A schematic diagram of Pyroptosis pathways. Pyroptosis is initiated when DAMPs or PAMPs activate the inflammasomes. Activated inflammasomes can lead to the cleavage of caspase-1. The activated caspase-1 cleaves Gasdermin D (GSDMD), in turn to form the N-fragment of GSDMD and cell membrane pores, resulting in pyroptosis. Activated caspase-1 also promotes the maturation and secretion of IL-1β and IL-18, which is also an important molecular event during the pyroptosis procession. When LPS binds to the precursor of caspase-4/5/11, it can also cause pyroptosis. The other way to activate pyroptosis is caspase-3/ Gasdermin E (GSDME). Caspase-3 can be activated by mitochondrial and death receptor pathway. The activated caspase-3 then cleaves GSDME, to produce GSDME N-fragments, forming pores in the plasma membrane, causing cell swelling and pyroptosis. DAMPs, danger-associated molecular patterns; PAMPs, pathogen-associated molecular patterns; IL-1β/IL-18, interleukin-1β/interleukin-18; LPS, lipopolysaccharide.

The pyroptosis can be induced through the canonical caspase-1 inflammasome pathways (17, 18) and non-canonical caspase-4/5/11 (caspase-4/5 in human and caspase-11 in mice) inflammasome pathways (19) (Figure 1). In canonical pyroptosis, a range of pathogen-associated molecular patterns (PAMPs) or danger-associated molecular patterns (DAMPs), including bacterial peptidoglycans, adenosine triphosphate (ATP), viral dsRNA, and the elevated intracellular reactive oxygen species (ROS) level (20–27), activate inflammasomes, such as absent in melanoma 2 (AIM2) (28), Pyrin (29), and the nucleotide-binding oligomerization domain (NOD)-like receptor (NLR) family (16, 30–32). In response to the inflammasomes stimulation, enzyme caspase-1 is recruited to the protein complex, which includes the inflammasome sensor itself, adaptor protein apoptosis-associated speck like proteins (ASC), and caspase activation and recruitment domain (CARD) of ASC, facilitating dimerization and activation (33). The activated caspase-1 is then capable of leading to maturation and secretion of IL-1β and IL-18. At the same time, the activated caspase-1 also cleaves gasdermin D (GSDMD) into two fragments: the N-terminal domain and C-terminal domain. The N-terminal fragment translocates to the inner leaflet of the plasma membrane and forms membrane pores with an inner diameter of 10–15 nm (34, 35). The membrane pores further promote the discharge of inflammatory factors, cell swelling, membrane rupture, and eventually lead to pyroptosis.

In contrast to canonical pyroptosis, non-canonical pyroptosis is mediated by caspases-4/5 in human and caspase-11 in mice. Caspase-4/5 (caspase 11 in mice) trigger the activation of pyroptosis through direct recognition of cytosolic lipopolysaccharide (LPS) via CARD domain (36–38). After that, the GSDMD protein is cleaved, which induced membrane pore formation, IL-1β maturation and release, cell membrane rupture, and ultimately pyroptosis.

In addition to the caspase-1 in the canonical and caspase-4/5/11 in the non-canonical inflammasome pathways, recent studies demonstrated that pyroptosis could also be activated by other caspases. Transforming growth factor-β (TGF-β)-activated kinase 1 (TAK1), a key molecule in TGF-β-induced Smad-independent signaling pathways, has recently been shown to be crucial for the modulation of nuclear factor kappa-light-chain-enhancer of activated B cells (NF-κB) signaling pathway. The inhibition of TAK1 by small-molecule inhibitor or Yersinia effector protein YopJ elicits caspase-8 dependent cleavage of GSDMD, which subsequently results in pyroptotic cell death (39, 40). As an essential modulator of cell apoptosis, caspase-3 has recently been demonstrated exerting a critical role in pyroptosis induction. Wang et al. (41) showed that caspase-3 involved in the regulation of pyroptosis through cleaving gasdermin E (GSDME).

## Gasdermin Proteins in Pyroptosis

Gasdermins are a family of pore-forming proteins which participate in the activation of pyroptosis. This family contains six members in human [GSDMA-E and Pejvakin (PJVK)] and 10 members in mice (three homologs of GSDMA (GSDMA1–3), four homologs of GSDMC (GSDMC1–4) and one homolog each of GSDMD, GSDME and PJVK) (42). Apart from PJVK, all of these proteins consist of two conserved domains, N-terminal effector domain and the C-terminal inhibitory domain (35, 43). In the resting state, gasdermins oligomerization is maintained by the intramolecular binding between the N-terminal effector domain and C-terminal inhibitory domain. In the presence of various microbial and endogenous stimuli, gasdermins is cleaved by pyroptotic caspases, the N-terminal domain of certain gasdermins squeeze into the lipid components, form pores in the cell membrane and execute the pyroptosis induction role (34, 35, 44, 45).

GSDMD and GSDME are two molecules that are extensively studied in pyroptosis. GSDMD, which mainly expressed in the gastrointestinal tract and skin, is a 53-kDa protein located downstream of the pyroptotic caspases (46, 47). As mentioned previously, GSDMD is an executioner of pyroptosis, which can be cleaved by pyroptotic caspases and form the cellular membrane pores. In response to the stimulation, the GSDMD N-terminal domain can bind to phosphatidylinositol phosphates of the cell membrane (34, 35, 43, 45, 48, 49). And the binding could be further enhanced by the interaction of GSDMD N-terminal domain and phosphatidic acid, phosphatidylserine and resulted in pore formation, cellular osmotic pressure change, cell membrane lysis, and pyroptosis (34).

GSDME is generally expressed in the fetal cochlea, heart, and kidney (50, 51). Studies suggest that the mutations of GSDME is related to the non-syndromic hearing impairment (50, 52, 53). In the regulation of pyroptosis, GSDME could be triggered by caspase-3, an important effector in apoptosis process. Activated caspase-3 cleaves GSDME and forms the N- and C-terminal domains. The N-terminal fragment of GSDME activated by caspase-3 is similar to N-terminal domain of GSDMD, resulting in cell membrane pore formation and pyroptosis (41, 54). However, the role of GSDME in driving pyroptosis upon apoptotic stimulation has been challenged by several studies. In the process of mitochondrial apoptosis, it was found that GSDME was unnecessary for the channel formation. GSDME played a non-redundant role in macrophage cell lysis downstream of the ripoptosome (55). In line with this study, Tixeira et al. (56) found that GSDME was dispensable for the pyroptosis regulation of human T cells and monocytes. Lee et al. (57) demonstrated GSDME was not required for pyroptosis in caspase 1^−/−^ caspase 11^−/−^ bone marrow-derived macrophages treated with flagellin, cytochrome c or Fas ligand. These results suggest that GSDME-mediated pyroptosis may only occur under specific conditions and specific cell types. The same as the GSDME-mediated pyroptosis, GSDME-independent secondary necrosis may also have a role in pyroptosis regulation. This new kind of pyroptosis may represent an interesting new inflammatory pattern of cell death, which will require further exploration of the details of its mechanism and significance in inflammatory diseases.

## Pyroptosis in Cancer

### Activation of GSDMD-Mediated Pyroptosis in Cancer

**Figure 2:**
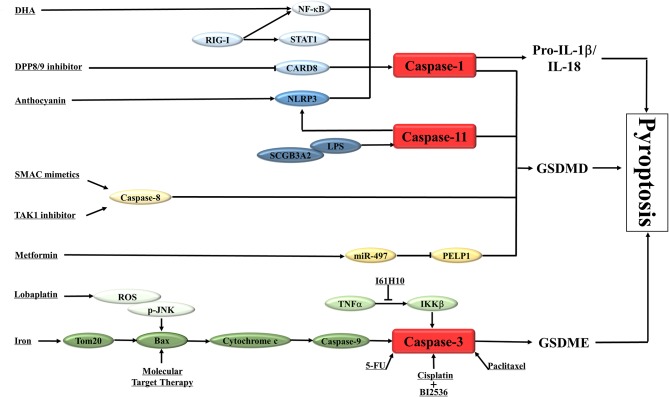
A schematic diagram shows the induction of pyroptosis in cancers. The main results of the studies on the activation of pyroptosis in cancers are summarized in the diagram. NLRP3, nod-like receptor protein 3; DDP8/9, dipeptidyl peptidase 8 and 9; CARD8, caspase activation and recruitment domain 8; RIG-I, retinoic acid inducible gene I; STAT1, signal transducer and activator of transcription 1; NF-κB, nuclear factor kappa-light-chain-enhancer of activated B cells; DHA, docosahexaenoic acid; IL-1β /IL-18, interleukin-1β/interleukin-18; GSDMD, gasdermin D; GSDME, gasdermin E; SCGB3A2, Secretoglobin (SCGB) 3A2; LPS, lipopolysaccharide; TNFα, Tumor necrosis factor α; IKKβ, inhibitor of nuclear factor kappa-B kinase subunit beta; Bax, Bcl-2-associated X protein; 5-FU, fluorouracil; SMAC, second mitochondrial-derived activator of caspases; TAK1, Transforming growth factor-β -activated kinase 1; PELP1, Proline-, glutamic acid- and leucine-rich protein-1; ROS, reactive oxygen species; JNK, c-Jun N-terminal kinase.

There are some studies indicated that certain drugs or molecules could trigger GSDMD-mediated pyroptosis in various types of cancer (Table 1; Figure 2), which suggested this new type of programmed cell death was involved in the pathogenesis of cancer and could be a new target in cancer management.

**Table 1:** Summary of the pyroptosis introductive reagents and the related cancer.

**Reagent**	**Cancer types**	**Mechanisms of pyroptosis activation**	**References**
Metformin	ESCC	miR-497/PELP1/GSDMD	Wang et al. (58)
Anthocyanin	OSCC	NLRP3/Caspase-1/IL-1β	Yue et al. (59)
DHA	Breast Cancer	NF-κB/Caspase-1/GSDMD	Pizato et al. (60)
DPP8/9 Inhibitor	AML	CARD8/Caspase-1/GSDMD	Johnson et al. (61)
α-NETA	Ovarian cancer	GSDMD	Qiao et al. (62)
Cisplatin; Paclitaxel	Lung cancer	Caspase-3/GSDME	Zhang et al. (63)
Iron	Melanoma	Tom 20/Bax/Cytochrome c/Caspase-9/Caspase-3/GSDME	Zhou et al. (64)
L61H10	Lung cancer	Cell Cycle Arrest/ NF-κB /GSDME	Chen et al. (65)
BI2536 and Cisplatin	ESCC	Caspase-3/GSDME	Wu et al. (66)
Lobaplatin	Colon cancer	ROS and JNK phosphorylation/ Bax/Cytochrome c/Caspase-9/Caspase-3/GSDME	Yu et al. (67)
Doxorubicin	Melanoma	eEF-2K/GSDME	Yu et al. (68)

*ESCC, esophageal squamous cell carcinoma; PELP1, Proline-, glutamic acid- and leucine-rich protein-1; GSDMD, gasdermin D; OSCC, oral squamous cell carcinoma; NLRP3, nod-like receptor protein 3; IL-1β, interleukin-1β; DHA, docosahexaenoic acid; DDP8/9, dipeptidyl peptidase 8 and 9; CARD8, caspase activation and recruitment domain 8; α-NETA, 2-(Anaphthoyl)ethyltrimethylammonium iodide; Bax, Bcl-2-associated X protein; ROS, reactive oxygen species; JNK, c-Jun N-terminal kinase; eEF-2K, eukaryotic elongation factor-2 kinase*.

Metformin is a widely used anti-diabetic drug. There are a great deal of evidences that metformin also have anti-cancer properties (69, 70). However, the exact mechanisms of the anti-cancer effect of metformin are still not completely clear. Recently, Wang et al. (58) showed that metformin could induce the GSDMD-mediated pyroptosis of esophageal squamous cell carcinoma (ESCC) in *in vitro* and *in vivo* studies. Mechanistic studies revealed that metformin activated the pyroptosis of ESCC by targeting miR-497/ Proline-, glutamic acid- and leucine-rich protein-1 (PELP1) pathway. These data clarify the exact mechanism of metformin-induced pyroptosis of cancer cells and provide an opportunity for the development of new drugs to control ESCC.

Anthocyanin is a kind of water-soluble natural pigment, which widely exists in plants and belongs to flavonoids. In recent years, more and more studies have revealed the therapeutic effect of anthocyanins in cancers (71). Yue et al. (59) investigated the potential inhibitory effect and underlie molecular mechanisms of anthocyanin on oral squamous cell carcinoma (OSCC). They noted that anthocyanin decreased the survival rate of OSCC cells and inhibited the migration and invasion of these cells via pyroptosis activation. The activation of pyroptosis was related to the increased expression of NLRP3, caspase-1 and IL-1β. Under the action of caspase-1 inhibitor, anthocyanin-activated pyroptosis was inhibited, and the cell survival rate, migration and invasion rate were increased.

Docosahexaenoic acid (DHA) is an omega-3 fatty acid with cancer inhibitory effect (72, 73). DHA inhibits the growth of breast cancer cells through NF-κB translocation and caspase-1 activation, which further cleaves GSDMD, promotes the secretion of IL-1 β, forms membrane pores, and leads to pyroptosis (60).

Dipeptidyl peptidase 8 and 9 (DPP8/9) are two relatively new members of the dipeptidyl peptidase IV family. Their role in regulating immune response and tumor proliferation has been reported before (74, 75). Johnson et al. (61) found that DPP8/9 inhibitor can induce pyroptosis in acute myeloid leukemia (AML). As an activator of “inflammasome” sensor CARD8, DPP8/9 inhibitor stimulates CARD8 dependent activation of caspase-1. Activated caspase-1 then leads to pyroptotic programmed cell death in AML. These results highlight the potential value of this small molecule DPP8/9 inhibitor in the treatment of AML.

In a recent study, Chen et al. (55) revealed that chemotherapeutic drugs such as the second mitochondrial-derived activator of caspases (SMAC) mimetics, TAK1 inhibitors and B-cell lymphoma 2 (Bcl-2) Homology 3 (BH3) mimetics could promote caspase-8 or caspase-9-dependent myeloid cells death. Activated Caspase-8 or caspase-9 could further cleave GSDMD, to promote the pyroptosis. These results suggest that innate immune cells have the ability to produce secondary necrosis signals in chemotherapy-induced apoptosis and play a new role in the regulation of cancer cell death.

Secretoglobin (SCGB) 3A2 (SCGB3A2) is a member of SCGB family, which has the function of anti-fibrotic (76). However, the role of SCGB3A2 in cancer development is unknown. In a recent study, the authors found that SCGB3A2, worked as a chaperone, directly interacted with syndecan-1 (SDC1) to facilitate the delivery of lipopolysaccharide (LPS) into the cytosol and stimulated pyroptosis in Lewis lung carcinoma (LLC) cells via up-regulating the caspase-11/NLRP3 pathway. *In vivo*, the role of SDC1 in SCGB3A2-mediated inhibition of growth and metastasis of LLC cells was also evaluated. In addition to the change of tumor volume, tumor metastasis was also affected by the interaction between SCGB3A2 and SDC1. The results showed that SCGB3A2 combined with LLC-sh-Control cells could significantly reduce the number of metastasis tumors, while the number of tumors in mice receiving LLC-sh-SDC1 cells and SCGB3A2 had no significant change compared with the control group. Mechanism studies demonstrated that the interaction between caspase-11 and SDC1 triggered the activation of non-canonical inflammasome pathway and pyroptosis. The effect of SCGB3A2 on the survival of lung-specific Kras^G12D^ mutant mice was also evaluated. Based on these results, the authors propose a new model, that is, SCGB3A2 chaperoned LPS to the cytosol through SDC1 and then lead to the pyroptosis of cancer cells driven by caspase-11 (77).

Retinoic acid inducible gene I (RIG-I) is the innate immune system pattern recognition receptor (PRRs), which plays a key role in RNA virus recognition (78, 79). It was found that the activation of RIG-I increased the number of tumor lymphocytes and reduced tumor growth and metastasis in breast cancer. The up-regulation of RIGI could stimulate the expression of pro-inflammatory transcription factors signal transducer and activator of transcription 1 (STAT1) and NF-κB. The activation of STAT1 and NF-κB further triggered exogenous apoptosis and caspase-1/GSDMD-regulated pyroptosis, accompanied by lymphocyte-recruiting chemokines and type I interferon release (80).

Resistance to chemotherapy is a major challenge for patients with ovarian cancer. It is equally important to clarify the mechanism of chemotherapy resistance in ovarian cancer as well as to develop new drugs for this kind of cancer. Recently, it was reported that 2-(Anaphthoyl)ethyltrimethylammonium iodide (α-NETA), an reversible choline acetylcholine transferase inhibitor, could inhibit the proliferation of epithelial ovarian cancer cell by inducing GSDMD/caspase-4 mediated pyroptosis. These results have also been confirmed in *in vivo* studies. Taken together, these findings suggest that induction of pyroptosis represents a new anticancer strategy for epithelial ovarian cancer therapy (62).

Long non-coding ribonucleic acid (LncRNAs) is also involved in the regulation of pyroptosis. Ma et al. (81) showed that inhibition of IncRNARP1-85F18.6 could promote the pyroptosis of colorectal cancer cells by modulating the expression level of ΔNp63. In addition, IncRNARP1-85F18.6, ΔNp63, and GSDMD also have certain value in the prognosis and diagnosis of colorectal cancer.

### Activation of GSDME-Mediated Pyroptosis in Cancer

Unlike GDSMD-medicated pyroptosis, GSDME-mediated pyroptosis is usually caused by chemotherapy agents or target therapy drugs (Table 1; Figure 2).

Wang et al. (41) found the different expression level of GSDME between cancer cells and many normal tissues. The GSDME-mediated pyroptosis could be activated by chemotherapy-induced caspase-3 activation in cancer cells. After chemotherapy, activated caspase-3 cleaves GSDME, to produce GSDME-N fragment, which can penetrate the cell membrane to induce pyroptosis. In addition, the low incidence of normal tissue damage and weight loss induced by chemotherapy was found in GSDME^−/−^ mice. Consistent with this, Zhang et al. (63) demonstrated that both cisplatin and paclitaxel could induced caspase-3/GSDME-mediated pyroptosis in lung cancer cells. However, the ability of cisplatin to induce pyroptosis was significant stronger than that of paclitaxel.

Iron is an important factor in cell regulation and body homeostasis (82, 83). A recent study has shown the amplification effect of iron on ROS and its role in the activation of pyroptosis in melanoma cells. In melanoma cells, iron can significantly enhance ROS triggered by chemotherapy, resulting in oxidation and oligomerization of mitochondrial import receptor subunit Tom20. Activated Tom20 recruits Bax to mitochondria and causes cytochrome c leakage. Cytochrome c further activates caspase-3, and eventually induces the cleavage of GSDME and the pyroptotic death of melanoma cells. To further verify the role of pyroptosis in the anti-tumor effect, A375 cells and GSDME-knockdown A375 cells were separately used to generate xenograft tumors in nude mice. Compared with the control group, sulfasalazine/ iron dextran solution treatment significantly decreased the tumor growth of A375 cells, at the same time, the results also showed that the GSDME cleavage increased. However, after the GSDME was knocked down, the efficacy of sulfasalazine/ iron dextran solution in the treatment of xenograft tumors was reduced. It suggested that iron could sensitize melanoma cells to ROS-induced drugs through GSDME and pyroptosis modulated pathways. In addition, because iron could amplify ROS to induce pyroptosis, it may be a potential sensitizer for melanoma treatment, which could induce the pyroptosis function of chemotherapeutic agents. In another words, the *in vivo* study suggested that the induction of pyroptosis might be a valuable approach to increase the efficacy of cancers (64).

Compound L61H10 is a heterocyclic ketone derivative, which has a role in cancer treatment. Study showed that L61H10 exerted the cancer inhibitory effects through arresting the cell cycle in the G2/M phase and mediating the NF-κB modulated apoptosis to GSDME-mediated pyroptosis transformation (65).

Serine/threonine protein kinase Polo-like Kinase 1(PLK1) plays an important role in the key steps of mitosis. PLK1 can inhibit DNA damage by inactivating e ataxia telangiectasia mutated and Rad3 related (ATR)/checkpoint kinase 1 (CHK1) pathway and ataxia telangiectasia-mutated gene (ATM)/CHK2 pathway (84, 85). Wu et al. (66) found that the combination of low dose PLK1 inhibitor BI2536 and cisplatin could induce pyroptosis in ESCC and increase the sensitivity of chemotherapy. In terms of mechanism, the pyroptosis introductive effect of BI2536 and cisplatin on ESCC cells depends on the caspase-3/GSDME axis. *In vivo*, the results showed that the tumor was successfully controlled according to the combined treatment of BI2536 and DDP. In addition, Wu et al. (66) showed that the co-treatment of BI2536 and DDP could induce the expression of cleaved caspase-3 and accumulate GSDME around the cytoplasm, resulting in pyroptosis of ESCC cells. It is further confirmed that BI2536 and DDP could induce pyroptosis through Bax/caspase-3/GSDME pathway. Furthermore, the prognostic role of GSDME in ESCC patients has also been confirmed. These results point to the importance of pyroptosis induction in cancer treatment, which may have an impact on clinical practice in the near future. However, the *in vivo* consequences of this cellular mechanism must be verified in order to predict whether the combination of BI2536 and DPP is an effective choice for cancer patients (86).

It was found that lobaplatin could induce the pyroptosis death of colon cancer cells by cleaving GSDME with caspase 3 in a recent study (67). The down-regulation of GSDME could change the cell death induced by lobaplatin from pyroptosis to apoptosis. In the mechanism study, the authors further found that lobaplatin elevated the level of ROS and c-Jun N-terminal kinase (JNK) phosphorylation. Activated JNK recruited Bax to mitochondria, which promoted the release of cytochrome c into the cytosol, and then induced caspase-3/-9 cleavage and pyroptosis. This study shows that GSDME-dependent pyroptosis is novel mechanism for the eradication of colon cancer cells by lobaplatin, which may be of great significance for the clinical application of anti-cancer therapeutics.

As a negative regulator of protein synthesis, eukaryotic elongation factor-2 kinase (eEF-2K) exerts a critical role in the regulation of autophagy and apoptosis in cancer cells (87, 88). In a recent study, the results revealed that eEF-2K also played an important role in pyroptosis of human melanoma cells induced by doxorubicin. Doxorubicin treatment could induce eEF-2K activation and autophagy in melanoma cells. However, eEF-2K silencing shifted the doxorubicin-induced autophagy into GSDME-modulated pyroptotic cell death, thus increasing the sensitivity of melanoma cells to doxorubicin. The author highlight that the results of the study provide new insight into cancer chemotherapy (68).

Recent study showed that pyroptosis was also involved in the response of target therapy in KRAS-, epidermal growth factor receptor (EGFR)—or anaplastic lymphoma kinase (ALK)-driven lung cancer. During target therapy, caspase-3-dependent GSDME activation can be triggered and lead to cytoplasmic membrane permeability and pyroptosis. This study provides a new idea for understanding the mechanism of targeted drugs and even the molecular mechanism of target therapy resistance (89).

Considering the potential role of pyroptosis in cancer treatment, some research teams are working to develop new anti-cancer drugs through induction of pyroptosis. Chalcone can increase the level of intracellular ROS and exert a wide range of biological activities in cancers (90, 91). Li et al. (92) incorporated α, β-unsaturated ketone unit into chalcone and developed a new compound. Compared with chalcone itself, the new compound has a better therapeutic effect on lung cancer. The therapeutic mechanism of this new compound may be related to the stimulation of caspase-3-mediated pyroptosis via elevating intracellular ROS levels. These findings lay a good foundation for the development of anti-cancer drugs based on induction of pyroptosis.

### Effects of Pyroptosis Introduction on the Cancer Immunity

The immunogenicity of cancer cells is a new determinant of anti-cancer immunotherapy. In addition to the development of dendritic cell-based vaccines, immune checkpoint inhibitors and adoptive T cell transfer, researchers have begun to pay more attention on the immunobiology of dead cancer cells and their correlation with the success of cancer immunotherapy (93, 94). Cell death is a basic biological phenomenon necessary for the survival and development of organisms. Recent evidence suggests that cell death contributes to immune defense against various types of disease (95). Pyroptosis is a programmed cell death pathway activated by several kinds of caspase, which can inhibit or increase the immunogenic potential of cancers. Physical rupture of pyroptotic cells leads to the release of pro-inflammatory cytokines IL-1β and IL-18, and endogenous DAMPs, indicating the immunogenic potential of pyroptosis (10). Additionally, the cytoplasmic contents of pyroptosis cells may be an effective signal to initiate inflammatory cascade. Recent studies have shown that IL-1β and IL-18 produced during pyroptosis can affect the recruitment of neutrophils (96). GSDMD, which is the most important effector of pyroptosis, could inhibit the response of cyclic GMP-AMP Synthase (cGAS)-driven type I interferon to cytoplasmic DNA and Francisella novicida in macrophages (97). In addition, GSDMD can also play an anti-inflammatory role by promoting neutrophil death (98). In a recent study, increased GSDMD cleavage was observed in OT-1 cytotoxic T lymphocytes (CTLs) and human activated CD8^+^ T cells. GSDMD also contributed to the cytolytic capacity of CD8^+^ T cells (99). These studies all support the immunogenic potential of pyroptosis. However, there is no direct evidence indicate that pyroptotic cell death can induce cancer immunity. A great deal of work needs to be done to further understand the role of pyroptosis in immunogenic in cancer.

### The Prognostic Role of GSDMD and GSDME in Cancer

The prognostic role of gasdermins, especially for GSDMD and GSDME, were demonstrated in recent studies.

Wang et al. (100) analyzed the role of GSDMD in the proliferation of gastric cancer. They found that GSDMD was down-regulated in gastric cancer and contributed to the occurrence and proliferation of this kind of cancer. Mechanistically, GSDMD inhibited extracellular signal-regulated kinase (ERK), STAT3, and phosphatidylinositol 3 kinase/protein kinase B (PI3K/AKT) signaling pathways in gastric cancer. Furthermore, the down-regulation of GSDMD also caused S to G2 cell cycle stage transition arrest via cyclin-dependent kinase 2 (CDK-2) and cyclin A2. These data indicated the tumor suppressor role of GSDMD in gastric cancer.

Oppositely, Gao et al. (101) reported GSDMD was up-regulated in non-small cell lung cancer. The high expression of GSDMD was related to larger tumor size, late tumor-node-metastasis (TNM) stages, and lower survival rate. However, the prognostic value of GSDMD was only found in lung adenocarcinoma, but not in squamous cell carcinoma. In subsequent studies, they found GSDMD induced lung cancer proliferation and poor prognosis through EGFR/AKT signaling pathway.

Unlike GSDMD, GSDME has been reported as a tumor suppressor in several studies. It was found that GSDME deficient melanoma cells formed and grew larger tumors than their wild-type counterparts (102). Furthermore, Wang et al. (103) revealed that GSDME could be cleaved by fluorouracil (5-FU) in a dose-dependent manner by activating caspase-3 in gastric cancer SGC-7901 and MKN-45 cells. Reversed the lower expression of GSDME in gastric cancer by decitabine could improve the efficacy of chemotherapeutic drugs. In addition, the positive correlation between GSDME mRNA level and the chemotherapy sensitivity of melanoma cells was also demonstrated in a recent study (104).

GSDMD and GSDME are two important pyroptotic substrates, and they also exert other critical roles in the pathogenesis and treatment strategies exploit of cancer. However, so far, we have not been able to conclude whether the level of gasdermin expression indicates a good or poor prognosis in cancers. We need more research to evaluate the prognostic role of Gasdermin in cancers.

## Conclusion

Pyroptosis, a new form of programmed cell death, has been widely studied in inflammatory disease models in recent years (11, 105). However, we are only just beginning to understand the molecular mechanisms of pyroptosis and its emerging role in cancer research (106). Although some studies have confirmed the critical role of pyroptosis in cancer, few cancer-specific mechanisms for the regulation of pyroptosis have been found. A great deal of work needs to be done to further understand the cancer specific regulation mechanisms of pyroptosis. The failure of treatment of some refractory cancers is largely due to the development of drug resistance to apoptosis. Therefore, introduction of non-apoptotic programmed cell death, such as pyroptosis, may be an effective way to rechallenge the apoptosis-resistant cancers (69). The identified new mechanisms in cancer cell pyroptosis may lead to the discovery of new drugs for cancer treatment in the future. In addition, because of the important role of GSDMD/GSDME in the regulation of both pyroptosis and cancer therapy sensitivity, the study which focus on GSDMD/GSDME and cancer treatment sensitivity will assign a new role for pyroptosis in the future.

## Author Contributions

Y-YW: conception and design. X-LL and RZ: manuscript writing. All authors: final approval of manuscript.

### Conflict of Interest

The authors declare that the research was conducted in the absence of any commercial or financial relationships that could be construed as a potential conflict of interest.
